# Ionic Liquid-assisted Synthesis of Celexocib Using Tris-(2-hydroxyethyl) Ammonium Acetate as an Efficient and Reusable Catalyst

**Published:** 2017

**Authors:** Hamid Reza Ahfad-Hosseini, Hasan Bagheri, Salimeh Amidi

**Affiliations:** a *Chemical Injuries Research Center, Baqiyatallah University of Medical Sciences, Tehran, Iran. *; b *Department of Medicinal Chemistry, School of Pharmacy, Shahid Beheshti University of Medical Sciences, Tehran, Iran.*

**Keywords:** Celecoxib, Anti-inflammatory agent, Ionic liquid, Trifluoroacetone, 4-methylbenzoylchloride, 4-hydrazinobenzenesulfonamide hydrochloride

## Abstract

Celecoxib is classified as none traditional nonsteroidal anti-inflammatory drugs (NSAIDs). This compound has conventional properties of NSAIDs such as anti-inflammatory, analgesic, and antipyretic activities beside reduced risk of gastrointestinal side effect of traditional NSAIDs such as ibuprofen. This compound gets a second sale rank of NSAIDs market at 2016 in the world and sales more than 17000 Kg in Iran during the past 6 month. So, a simple, rapid and green method for synthesis of this compound is important. In the present study, a novel green method was suggested for the synthesis of celecoxib using the ionic liquid.

Celecoxib was provided by the reaction of trifluoroacetone, 4-methylbenzoylchloride, and 4-hydrazinobenzenesulfonamide hydrochloride. The tris-(2-hydroxyethyl) ammonium acetate as ionic liquid was prepared by mixing tris-(2-hydroxyethyl) ammonium and acetic acid, and used as an efficient catalyst. The structure of the synthetic products was confirmed by analytical and spectroscopic methods including ^1^HNMR, ^13^CNMR, IR, MS and elemental analysis.

This ionic liquid can play dual roles in the synthesis of celecoxib, as a catalyst to improve electrophilicity of carbonyl group and also as a solvent of reaction. The reaction rate and yield (86%) were improved considerably. Moreover IL showed the same efficiency when used in 4 consecutive reactions.

## Introduction

Non-steroidal, anti-inflammatory drugs (NSAIDs) are a class of medications that possess analgesic and anti-pyretic activities (1). NSAIDs are perhaps the commonly used and avaliable medications today in healtheare services for diversified medical disorders. Routinly, these are used for reducing pain and inflammation in various musculoskeletal disorders and menstrual cramps (2). NSAIDs exert their pharmacological effects via inhibition of cyclooxygenase (COX) isoforms (COX-1 and COX-2) (3). 

Celecoxib is a selective COX-2 inhibitor and classified as none traditional nonsteroidal anti-inflammatory drugs (NSAIDs). This compound exhibits anti-inflammatory (4), analgesic (5), and antipyretic activities in animal models (6) and humans (3). The celecoxib mechanism of action is believed to be due to inhibition of prostaglandin synthesis, primarily via inhibition of COX-2, and at therapeutic concentrations in humans, it does not inhibit the COX-1 isoenzyme (7, 8). Beside conventional properties of NSAIDs, this compound has less gastrointestinal side effects when compared to other anti-inflammatory agents, notably non-steroidal anti-inflammatory drugs (NSAIDs) such as aspirin and ibuprofen, which inhibit both COX-1 and COX-2 (9, 10).

Moreover, discovery of the COX-2 role in the angiogenesis and cellular mechanism of cancer, reveal the new indication for selective COX-2 inhibitors in cancer chemotherapy and neurological diseases such as Parkinson and Alzheimer’s diseases. Since, celecoxib is a selective COX-2 inhibitor; it can have indication for these diseases as well as conventional uses. 

Industrial synthesis of pharmaceutical compounds often involves the use of organic solvents mainly for reasons of cost effective procedure and ease of handling. Unfortunately, these reaction media are responsible for organic contamination of the final product and are therefore referred to as ‘residual solvents’ or ‘organic volatile impurities’. The acceptable limits for contaminants resulting from the entire drug product manufacturing process have been specified in pharmacopoeias and the International Conference on Harmonization (ICH) of Technical Requirements for Registration of Pharmaceuticals for Human Use (11). The ICH guideline distinguishes four classes of residual solvents in drug substances: Solvents to be avoided, solvents to be limited, solvents with low toxic potential and solvents without adequate toxicological data. From the toxicological point of view, genotoxic impurities (GTIs) are the most dangerous contaminants for human health. Exposure to even low levels of such impurities present in the final active pharmaceutical ingredient (API) may induce genetic mutations and may potentially cause cancer in humans (12, 13). However, regardless of the solvent class, it is important to explore the possible opportunities to reduce or avoid the use of harmful solvents in the manufacturing process of pharmaceuticals. 

The use of ionic liquids (ILs) as non-conventional media in chemical synthesis has increasing attention because of their physical and chemical properties. Their growing application in organic chemistry stems from their favorable physicochemical properties, such as the lack of vapor pressure, excellent thermal and chemical stability and very good dissolution properties of both organic and inorganic compounds (14). The application of ‘green’ solvents, such as ionic liquids, in the pharmaceutical industry, is currently being extensively investigated at the laboratory scale (15, 16).

**Figure 1:**
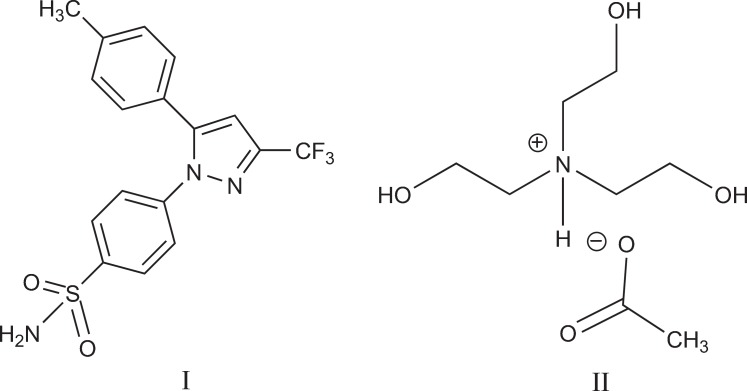
Structure of celecoxib (I) and tris-(2-hydroxyethyl)ammonium acetate (II)

Celecoxib (I) is chemically designated as 4-[5-(4-methylphenyl)-3-(trifluoromethyl)-1H-pyrazol-1-yl] benzenesulfonamide and is a diaryl-substituted pyrazole (Figure 1) (17-19). 

Celecoxib get a second sale rank of NSAIDs market at 2016 in the world (20) and more than 17000 Kg sales in Iran during the 6 month. So, the simple, rapid and green method for synthesis of this compound is important. Diverse methods have been used to synthesise celecoxib, and new derivatives of that since it was discovered, such as condensation reaction of 4-hydrazinylbenzenesulfonamide and 4,4,4-trifluoro-1-(p-tolyl) butane-1,3-dione which due to lack of regioselectivity the product is a mixture of pyrazole regioisomers so can be difficult to purify. The other methods are 1,4 addition and then condensation reaction of 4-hydrazinylbenzenesulfonamide and (E)-1,1,1-trifluoro-4-(p-tolyl)but-3-en-2-one or cycloaddition reaction of benzene sulfonic 2,2,2-trifluoro-N-(4-sulfamoylphenyl)acetol hydrazonic anhydride and 4-(1-(p-tolyl) vinyl) morpholine which in both methods the toxic solvent such as ethyl acetate, methanol etc., were used at reflux system with low yield (21, 22).

Seeking highly-efficient, low-cost and green methods to synthesise celecoxib is very much in demand. Here, we introduced a new method for synthesis of celecoxib with high yield and least damage to the environment by using *tris-(2-hydroxyethyl)ammonium acetate* (II) as ionic liquid (Figure 1) and evaluated the effect of IL concentration and reaction temparature on the yeild of celecoxib production.

## Material and methods

*General*

All reagents and solvents were purchased from Merck (Darmstadt, Germany) and Sigma-Aldrich Chemical Company. All of which were used without further purification. IR spectra were recorded by Perkin Elmer FT-IR spectrum Gx, using KBr pellets. The NMR spectra were recorded on a Jeol FT-NMR-400 MHz in DMSO solvent using TMS as an internal standard. Melting points were measured on an Electrothermal 9100 apparatus.

*Synthesis of Tris-(2-hydroxyethyl)ammonium acetate as ionic liquid (IL)*

**Scheme 1:**
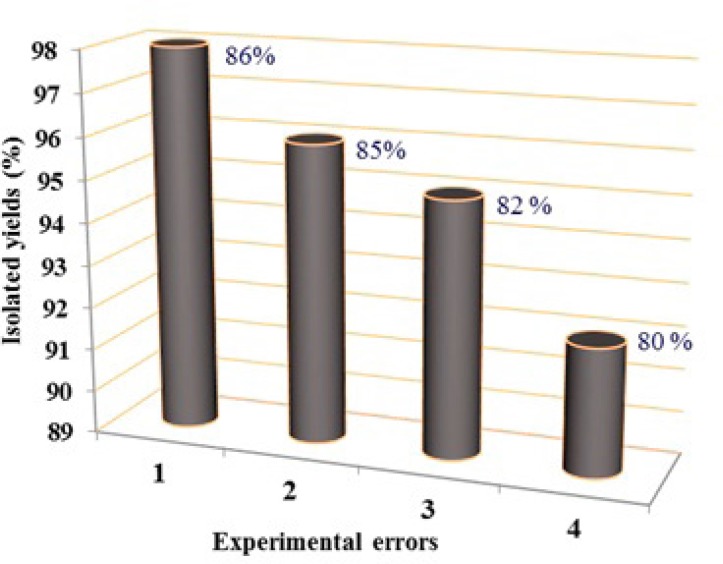
Synthesis of Tris-(2-hydroxyethyl)ammonium acetate as ionic liquid

As shown in scheme 1 the IL was prepared by adding 5 mmol of acetic acid and 5 mmol of triethanolamine to 10 mL dichloromethane with continuous stirring in room temperature for 2 h. The light brown solid powder appeared which was isolated by filtration, washed with diethyl ether and dried in vacuum oven.

*Tris-(2-hydroxyethyl)ammonium acetate*

Light brown, yield 100%, mp: 90 °C; IR (KBr): υ 3356, 3152, 300, 1686 cm^-1^, ^1^H NMR (400 MHz, DMSO-d_6_): δ-ppm 2.21 (s, 3H, —CH_3_ aliphatic), 3.4 (t, 6H, —CH_2_ aliphatic), 3.8 (t, 6H, —CH_2_ aliphatic), 4.7 (s, OH), 6.8 (s, N-H). 13C NMR (100 MHz, DMSO-d6): δppm 151, 55.2, 54.9, 21.4.

*Synthesis of celecoxib*

Two mmol of 1,1,1-trifluoroacetone, 2 mmol of 4-methylbenzoylchloride, and different amount of the IL (0.5 – 10 mol%) were dissolved in the mixture of water: ethanol (8 mL: 2 mL) with continuous stirring at room temperature. After half an hour, 2 mmol of 4-hydrazinobenzenesulfonamidehydrochloride was added to the mixture. White powder appeared which was isolated via filtration, washed with a mixture of water : ethanol (70:30) and then was dried in vacuum oven. 

*Celecoxib*

White powder, yield 86% (for 2 mol% of IL), mp: 125.1–126.1 °C; IR (KBr): υ 3337, 3000, 2800, 1650, 1590, 1500 cm^-1^, ^1^H NMR (400 MHz, DMSO-d6): δppm 2.6 (s, 3H, -CH3), 5.1 (s, 2H, -NH2), 6.5 (s,1H, ArH), 7.2-7.5 (m, 4H, ArH), 7.6-8.4 (m , 4H, ArH). ^13^C NMR (100 MHz, DMSO-d6): δppm 145, 143, 142, 135, 107. MS: [M+1]^ +^ *m/z* 382.4. Anal. Calcd. for C_17_H_14_F_3_N_3_O_2_S: C, 53.54; H, 3.70; N, 11.02. Found: C, 53.79; H, 3.65; N, 10.84. 

## Results and discussion

In the present work the simple, rapid and green method for synthesis of celecoxib with the ionic liquid was suggested. The structure of the ionic liquid and celecoxib were confirmed by appropriate spectroscopic methods such as ^1^H NMR, ^13^C NMR, IR and mass spectroscopy (MS). 

*Synthesis of IL*

*Tris-(2-hydroxyethyl)ammonium acetate* as the ionic liquid was synthesis by an efficient synthetic route outlined in the Scheme 1. 

In the ^1^H NMR spectrum, the peak appears at 2.21 ppm related to protons of CH_3_ of acetate while protons which are shown as 1 and 2 in Scheme 1, appeared at 3.4 and 3.8 ppm respectively. The singlet peak at 4.7 ppm dependent to OH and finally the singlet peak at 6.8 ppm which confirms the synthesis of IL, related to the proton of NH. The IR spectrum of IL showed absorption at 3152 cm^-1^ belongs to N-H, which confirms the synthesis of IL.

*Synthesis of celecoxib*

**Scheme 2:**
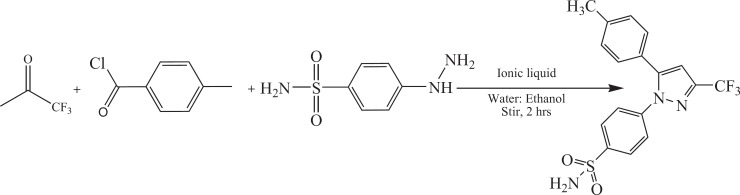
Synthesis of celecoxib

The synthetic route for celecoxib was outlined in the Scheme 2. The ^1^H NMR spectrum of synthesis celecoxib has sharp singlet peak at 2.6 ppm belong to 3 protons of methyl and another one at 5.1 ppm related to NH_2_ protons. The proton of pyrazole appears at 6.7 ppm as a singlet peak while the protons of aryl ring are observed as a broad peak at 7.2-7.5 ppm. The multiple peaks between 7.6-8.4 ppm belongs to protons of benzenesulfonamide ring. In ^13^CNMR spectrum of celecoxib, the peaks appeared at 145 and 143 ppm, related to carbons of pyrazole ring. These peaks confirm the reaction of trifluoroacetone, 4-methylbenzoylchloride, and 4-hydrazino benzene sulfonamide hydrochloride. Another carbon of pyrazole ring appeared at 107 ppm. Mass analysis was performed at unit resolution in the positive ion mode for celecoxib. The signal intensity for [M + H] ^+^ ion 4 was observed at m/z 382 which confirmed the formation of celecoxib. Important bonds were observed in IR spectrum of celecoxib, include: the absorption at 3337 cm^-1^ related to vibration of amine type 1 (N-H) and the bond at 2800-3000 cm^-1^ belong to vibration of C-H, Sharp absorptions at 1100 and 1300 cm^-1^ attributed to the presence two bands of S=O, also, Vibration of C=C appear at 1400-1500 cm^-1^.

**Figure 2:**
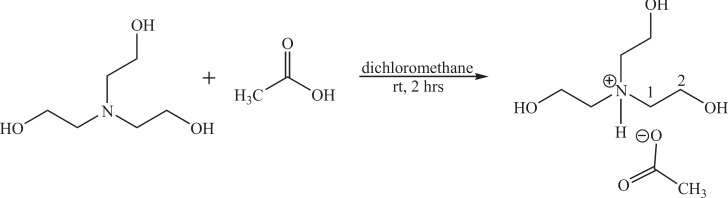
Study of IL reusability

**Scheme 3:**
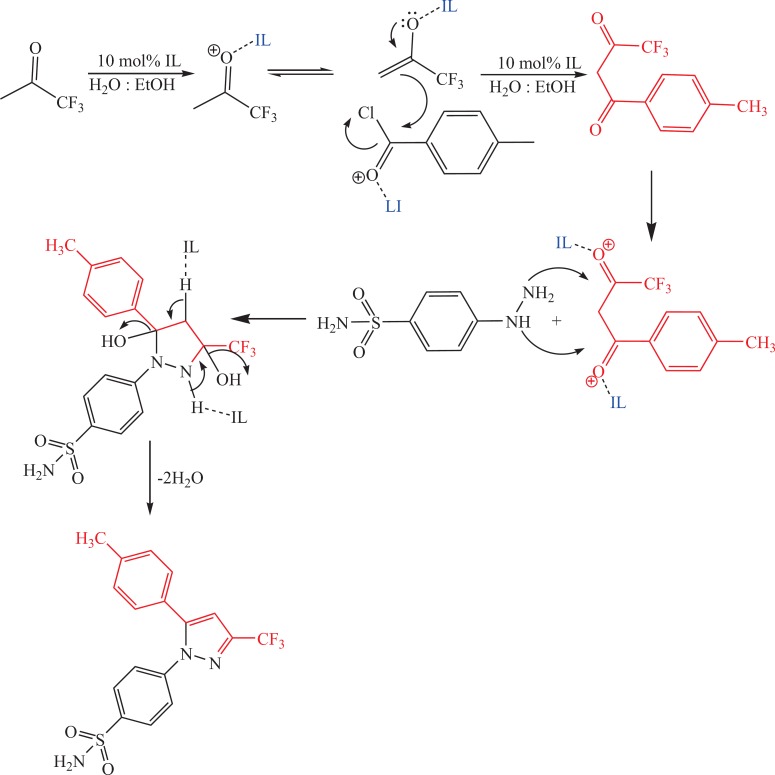
The suggested mechanism for the synthesis of celecoxib in the presence of ionic liquid.

*Mechanism of IL in the synthesis of celecoxib*

The suggested mechanism that illustrates the effect of IL on the trend of synthesis reaction of celecoxib is represented in Scheme 3.

*Effect of IL amount and temperature on reaction yield*

Effect of different amounts of the IL and temperature was investigated in yield of the reaction. The results were shown in Table 1. According to obtained results, 2 mole% of the IL at room temperature led to maximum yield of 86%. This yield was higher than a previously reported method by Lynette M Oh in 2006 (yield 72%) (22). Water was used for dissolving the IL.and increase solubility, stability of cation and better performance of the IL due to hydrogen bond formation with three OH groups of cation part of the IL. Also, ethanol improves solubility of reactants.

It should be noted that, at the end of the reaction, the formed product could be easily isolated with IL catalyst using water. The recovered IL could be reused in the next run after 20 min of treatment under vacuum conditions at 80 ^°^C (20 mmHg). The catalytic activity of the IL keeps almost unchanged in four runs; the results are shown in Figure 2.

**Table 1:** Effect of different amounts of the IL on celecoxib reaction's yield

Entry	Catalyst loading (mol %)	Reaction temperature (^o^C)	Reaction time (min)	Yield^b^ (%)
1	—	r.t.^1^	50	10
2	—	90	50	25
3	0.5	r.t.	50	45
4	0.5	90	50	60
5	1	r.t.	50	70
6	1	60	50	80
7	2	r.t	50	86
8	2	60	50	86
9	5	r.t	50	86
10	10	r.t	50	86

1- Room Temperature

## Conclusion

In summary, a novel, green and efficient salt namely tris-(2-hydroxyethyl) ammonium acetate was synthesized and characterized by IR, ^1^HNMR and ^13^CNMR techniques. The application of this salt in water as a solvent, at room temperature was considered in the synthesis of celecoxib by trifluoroacetone, 4-methylbenzoylchloride, and 4-hydrazinobenzenesulfonamide hydrochloride. The achieved results showed that the structure of IL plays a key role in the trend of the reaction of synthesic celecoxib and led to higher yields and shorter reaction time. Other significant advantages of this study are high yield, low cost, the simplicity of product isolation, reusability of the IL and compliance with the green chemistry protocols.
